# Long-Term Exposure to Microplastics Promotes Early-Stage Hepatocarcinogenesis Induced by Diethylnitrosamine in Rats by Modulation of Their Gut Microbiota

**DOI:** 10.3390/toxics13050353

**Published:** 2025-04-29

**Authors:** Huina Guo, Jianan Wang, Shaowen Huang, Suren Rao Sooranna, Fangyi Shu, Genliang Li

**Affiliations:** 1 School of Basic Medical Sciences, Youjiang Medical University for Nationalities, Baise 533000, China; wjnnt163@163.com (J.W.); shufangyi02@sina.com (F.S.); ligenliang@163.com (G.L.); 2 Department of Laboratory Medicine, Baise People’s Hospital, Baise 533000, China; huangshaowen2025@163.com; 3 Department of Metabolism, Digestion and Reproduction, Faculty of Medicine, Imperial College London, London SW10 9NH, UK; s.sooranna@imperial.ac.uk

**Keywords:** microplastics, rat, hepatocarcinogenesis, preneoplastic lesions, diethylnitrosamine, microbiota, short chain fatty acids

## Abstract

Hepatocarcinogenesis is linked to environmental factors, with microplastics (MPs) emerging as a global environmental concern that may contribute to liver injury. However, the impact of MPs on the early stages of hepatocarcinogenesis has been largely ignored. Here we investigated the impact of long-term MP exposure on the formation of preneoplastic lesions during hepatocarcinogenesis induced by diethylnitrosamine (DEN) in rats. Rats were injected with DEN to induce preneoplastic lesions, and then they were orally administered with 1 µm MPs 0.5 mg/kg body weight per day for 20 weeks. The results revealed that long-term exposure to MPs did not induce the formation of glutathione *S*-transferase placental form (GST-P)-positive foci as preneoplastic lesions during hepatocarcinogenesis in these animals, thereby indicating non-carcinogenicity. However, MP exposure resulted in a 1-fold increase in both the number and size of GST-P-positive foci in rats initiated with DEN compared to those treated with DEN alone. Accordingly, MP exposure led to a 0.61-fold increase in the index of proliferating cell nuclear antigen (PCNA)-positive cells in DEN-initiated rats when compared to DEN treatment alone. In addition, the composition of the gut microbiota was significantly altered, accompanied by various levels of short-chain fatty acids. Our results suggest that long-term MP exposure can promote pre-neoplastic lesion formation in DEN-induced rats by increased cell proliferation as well as alterations in the gut microbiota and short-chain fatty acid levels. This highlights the potential health risks associated with hepatocarcinogenesis linked to long-term exposure to MPs.

## 1. Introduction

Microplastics (MPs) are plastic particles less than 5 mm, originating from either manufacturing processes or the breakdown of larger plastic debris. They have emerged as a significant global environmental issue due to the huge increase in annual emissions, their ubiquity and their persistence in worldwide ecosystems [1]. Humans are inevitably exposed to MPs via the food chain and when they are inhaled [2]. Plastic particles with a diameter of less than 3 µm are more easily ingested, and these exhibit more diverse and adverse effects on organisms [3]. To date, MPs have been detected in feces, blood, placenta, liver, kidney and lungs, indicating both their potential health risks and their abilities to traverse, accumulate and be excreted by the human body [4]. Related toxicological investigations of MPs have steadily increased, encompassing over 75 animal studies [5].

Hepatocarcinogenesis is a complex, multiple process influenced by environmental factors including cigarette smoke, alcohol, aflatoxin B, viruses and so on [6]. Moreover, liver cancer is the third leading cause of cancer-related deaths globally [7]. Integrated analysis of multinational registry data has revealed that liver cancer exhibits the highest correlation coefficient among cancers related to MP pollution [8]. Notably, MPs have been detected in cirrhotic liver tissues but not in those from healthy donors [9]. Traversa et al. found that acute polyethylene micro/nanoplastic (PE-MNPs) exposure induced cytoskeletal changes and epithelial mesenchymal transition (EMT)-like phenotypes in lung cells, promoting cell migration without overt toxicity [10]. This finding suggests that MNPs may have a potential role in early carcinogenesis. Therefore, it is essential to evaluate the impact of MPs on the development of hepatocarcinogenesis. However, the impact of MPs on humans suffering from hepatocarcinogenesis has not been well studied. The liver plays a critical role in detoxification in mammals, which possesses the capacity of self-defense against xenobiotic attacks. Reports indicate that MPs can induce mice liver damage through oxidative stress, an inflammatory response, metabolism disorder and gut microbiota alteration [11,12,13]. Additionally, MPs exacerbated liver injury in mice induced by drugs such as acetaminophen and cyclophosphamide [14,15]. These studies suggest that liver resistance might be attenuated in a sub-healthy state, leading to the aggravation of liver injury. Thus, we hypothesized that MPs could promote the development of hepatocarcinogenesis.

Given the anatomic and functional link between the gut and liver, there is increasing evidence highlighting the crucial role of the gut microbiota in hepatocarcinogenesis [16,17]. The underlying mechanisms may involve microbiota dysbiosis, metabolite production and immune disorders that occur between the host and the gut [17]. Gut dysbiosis is characterized by alterations in the composition and function of the gut microbiota due to an imbalance between beneficial and harmful microorganisms [18]. Ni et al. recruited patients with primary hepatocellular carcinoma (HCC) and healthy controls, and they demonstrated a significant increase in pro-inflammatory bacteria, specifically *proteobacteria*, in HCC patients compared to healthy controls [19,20]. In addition, the microbiota can produce a variety of metabolites, such as short-chain fatty acids (SCFAs). SCFAs, including acetate, propionate and butyrate, are generated through the fermentation of dietary fibers by the gut microbiota, and they have been shown to exhibit anti-inflammatory, antioxidant and anticancer properties [21]. MPs could induce gut microbiota dysbiosis and hepatic lipid metabolism disorder in both mice and human liver organoids [11,22]. Given the potential of MPs to disrupt the gut microbiota and its metabolite production, it is essential to investigate whether MPs could affect hepatocarcinogenesis by modulating the gut microbiota and SCFAs levels. Understanding these relationships may provide new insights into the environmental factors contributing to liver cancer development. This will give law enforcement agencies crucial evidence to promote the reduction of potentially harmful substances in our environment.

There is a lack of studies evaluating the impact of MPs on the development of hepatocarcinogenesis. Consequently, in this study, diethylnitrosamine (DEN), a well-known chemical carcinogen, was utilized to induce the early stages of hepatocarcinogenesis in rats. Furthermore, Pontecorvi et al. demonstrated that MPs induce acute toxicity at high concentrations, but they allowed for recovery during chronic exposure in vaginal keratinocytes. In all instances, exposure to MPs resulted in persistent epigenetic alterations, highlighting the necessity for evaluating their long-term effects [23]. Several studies have focused on the duration of MP exposure in mice, which typically ranged from a few days to three months [24,25], and it was shown that the effects of MPs on organisms is time-dependent effect [13]. Therefore, we evaluated the impact of long-term exposure to MPs on the formation of preneoplastic lesions during the early stages of hepatocarcinogenesis induced by DEN in rats, with a particular emphasis on the role of the gut microbiota.

## 2. Materials and Methods

### 2.1. Animals and Experimental Protocol

#### 2.1.1. Animals and Exposure

The animal protocol was approved by the Animal Care Committee of Youjiang Medical University for Nationalities (Ethics Number: 2023032901). Male 3-week-old Wistar rats (90–100 g) were purchased from Vital River Laboratory Animal Technology Co. Ltd., Beijing, China, under the laboratory animal certificate number 20230329Aazz0600000752. The age of rats was chosen based on previous studies, and this was considered a time point when the animals were more sensitive to the effects of carcinogens [26]. All rats were maintained at 25 ± 1 °C under a 12-h light–dark cycle and provided with water and a standard diet. Rats were group-housed (3–4 animals/cage) in cages with sterile corn cob bedding, which was changed every 48 h. After 1 week of adaptation, animal studies were initiated by following the relevant regulations and guidelines.

#### 2.1.2. Experimental Design

**Figure 1:**
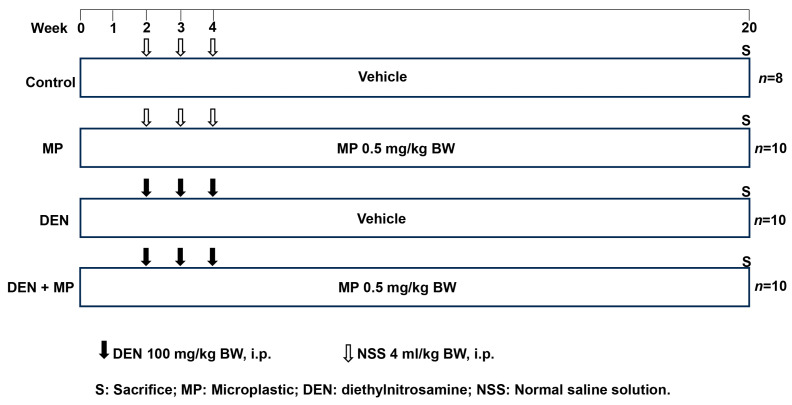
The animal protocol for microplastics administration in DEN-induced preneoplastic lessons in the livers of rats. BW, body weight; i.p., intraperitoneal injection.

Rats were randomly assigned into four groups: Control, MP, DEN and DEN + MP. The protocol details are illustrated in Figure 1. Each group included 10 rats, with the exception of the control group (*n* = 8). The Control and DEN groups served as negative and positive controls, respectively. The MP and DEN + MP groups allowed for the evaluation of both the individual and interactive effects of MPs and those of carcinogen exposure. The Control and DEN groups were orally administered with distilled water, while the MP and DEN + MP groups were oral gavage with a relatively low dose of 0.5 mg of polystyrene MPs (PSMPs) per kg of body weight throughout the 20-week experiment. Daily gavage at a fixed time of 9:00 am. PSMPs (1 μm, 1.064 g/cm^3^, 25 mg/mL) have been well characterized by using transmission electron microscopy [11] and were obtained from this manufacturer. Prior to use, the PSMPs were added to distilled water and sonicated for 30 min to ensure complete suspension. The exposure dose of PSMPs was calculated based on previous studies, considering that humans can intake 10^10^ items of MPs per day if they drink one teabag and in comparison with the doses used in other studies [27,28,29]. The DEN and DEN + MP groups were injected with 100 mg/kg body weight (BW) of DEN during weeks 2, 3 and 4 to induce hepatic preneoplastic lesion formation. The Control and MP groups were intraperitoneally injected with a normal saline solution. DEN was purchased from GlpBio (GC19566, GlpBio, Montclair, CA, USA). The general observations included food and water intake levels, as well as the BWs of the rats, which were recorded twice weekly. At the end of the experiment, all rats were euthanized via inhalation of 4% isoflurane. Blood samples were collected to assess alanine aminotransferase (ALT) and aspartate aminotransferase (AST) levels using enzyme kinetics methods, as specified by the manufacturer (C009-3-2/C010-3-1, Nanjing Jiancheng Bioengineering Institute, Nanjing, China). The livers, spleens and kidneys of rats were excised, and then they were weighed. Three pieces of liver tissue were fixed in 10% phosphate-buffered formalin and embedded in paraffin wax. In addition, fecal samples were collected from the anus, and these were packed in dry ice and sent for microbiota and SCFAs analyses at Majorbio Bio-Pharm Technology Co., Ltd. (Shanghai, China).

### 2.2. Determination of Liver Preneoplastic Lesions and Cell Proliferation

The investigation of preneoplastic lesions in the livers induced by DEN in rats was conducted using immunohistochemical staining with the anti-GST-P antibody (311, MBL, Nagoya, Japan), as detailed in our earlier studies [30]. The liver sections were deparaffinized and dehydrated, then immersed in 3% hydrogen peroxide and 1% skimmed milk. Subsequently, the sections were incubated with a polyclonal rabbit anti-rat GST-P antibody (1:1000). Following this, the sections were incubated with a secondary antibody (goat anti-rabbit IgG) (BA1003, Boster Biological Technology, Wuhan, Hubei, China), which was conjugated with an avidin–biotin–peroxidase complex-based amplification system (ABC-PO kit) (PK-4000, Vector Laboratories, Burlingame, CA, USA). This process resulted in the development of a brown color using diaminobenzidine (DAB) (AR1000, Boster Biological Technology, Wuhan, China). Finally, the sections were counterstained with hematoxylin. The brown GST-P-positive foci were observed and captured under a normal microscope (BA210, Motic, Xiamen, China). The whole areas of liver tissues were captured by using an Automated Digital Slide Scanning System (VM1000, Motic, Xiamen, China), and we analyzed the areas with the ImageJ program (1.53, NIH, Bethesda, MD, USA). The numbers and areas of brown GST-P-positive foci greater than 0.16 mm^2^ were used for statistical analysis [31]. The results were expressed as the areas and numbers of GST-P-positive foci for each liver area examined.

Proliferating cell nuclear antigen (PCNA) was used as a cell proliferation marker. The prepared sections were initially incubated with a monoclonal mouse anti-rat PCNA antibody (633504, BioLegend, San Diego, CA, USA), followed by incubation with a biotinylated goat anti-mouse IgG antibody (BA1001, Boster Biological Technology, Wuhan, China). The sections were then incubated with an ABC-PO kit, and color development was achieved with DAB. The staining protocols used were detailed by Phannasorn et al. [32]. The PCNA-positive cells were counted in 10 randomly selected fields per slide at 100× magnification using a light microscope. The results were represented as PCNA-positive cell per field.

### 2.3. Investigation of Fecal Intestinal Microbiota in Rats

The quality and quantity of extracted fecal genomic DNA was assessed by 1% agarose gel electrophoresis and measuring the OD260/280 ratio. Hypervariable regions V3–V4 of the bacterial 16S rRNA gene were amplified using the forward primer 338 (5′-ACT CCT ACGGGA GGC AGC AG-3′) and the reverse primer 806 (5′-GGA CTA CHVGGG TWT CTAAT-3′) on an ABI GeneAmp^®^ 9700 PCR thermocycler. Subsequently, all PCR products were extracted, purified and quantified to obtain purified amplicons. These purified amplicons were then constructed and sequenced on an Illumina MiSeq platform (Illumina, San Diego, CA, USA) by Majorbio Bio-Pharm Technology Co. Ltd. (Shanghai, China) [33]. The raw data underwent processes of data splitting, quality control and assembly to obtain optimized datasets. De-noising was performed using the divisive amplicon denoising algorithm 2 (DADA2) to produce the final, accurate biological sequences, referred to as amplicon sequence variants (ASVs) [34]. The analysis of 16S rRNA microbiome sequencing data was conducted using the Majorbio Cloud Platform (https://www.majorbio.com) (accessed on 30 November 2023).

### 2.4. Measurement of SCFAs in Rat Feces

SCFAs including acetic, propanoic, butanoic, isobutyric, valeric, isovaleric, hexanoic and isohexanoic acids were quantified in fecal samples by using the manufacturer’s protocol (Majorbio Bio-PharmTechnology Co., Ltd., Shanghai, China). Briefly, the fecal samples (20 mg) were extracted with 800 μL of 0.5% phosphate water, which contained 10 µg/mL 2-ethylbutyric acid as an internal standard, following standard protocols. Gas chromatography–mass spectrometry (GC–MS) analysis was performed by using an Agilent 8890B–5977 B GC–MS system equipped with an Agilent HP FFAP capillary column (30 m × 0.25 mm × 0.25 μm) [35]. Data acquisition was conducted in full-scan mode, and the compounds were identified and quantified using the Masshunter software (v10.0.707.0, Agilent Technologies, Santa Clara, CA, USA). The amounts of SCFAs were calculated from a mixed standard curve, and these were expressed as μg/mg of feces.

### 2.5. Statistical Analysis

The data are presented as mean ± SD values. After confirming normality using the Shapiro–Wilk test (*p* > 0.05), a one-way analysis of variance (ANOVA) with the least significant difference (LSD) *post hoc* test was performed for multiple comparisons. For 16S rRNA analysis, multiple-group comparisons were performed using the Kruskal–Wallis H test by applying a false discovery rate (FDR) correction. In all cases, a difference of *p* < 0.05 was considered to be statistically significant. All possible pairwise comparisons between the study groups were performed.

## 3. Results

### 3.1. MP Altered the Growth Phenotypes and Liver Enzyme Levels in DEN-Induced Rats

Based on the general observations presented in Table 1, the administration of MP and DEN injection alone did not significantly affect BWs or daily consumption in rats. However, long-term administration of MP in DEN-initiated rats caused a significant decrease in BWs, as well as in the relative weights of the livers and spleens (Table 1 and Table 2). Additionally, the liver function enzyme, ALT, was significantly elevated in the DEN + MP group compared to both the Control and DEN groups. In contrast, the liver function enzyme levels were not altered in the DEN and MP groups. These findings indicated that MP and DEN exhibited a synergistic adverse effect in rats.

### 3.2. MP Promoted Preneoplastic Lesion Formation in the Livers of DEN-Induced Rats

**Figure 2:**
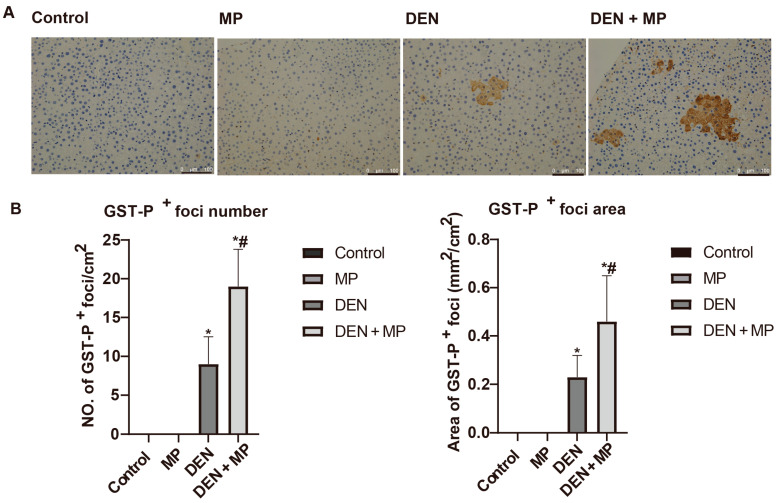
The effects of microplastics administration on DEN-induced preneoplastic lessons in livers of rats. (**A**) GST-P-positive foci in rat livers, (**B**) the number and areas of GST-P-positive foci in rats. Glutathione *S*-transferase placental form (GST-P), Microplastic (MP), Diethylnitrosamine (DEN). The scale bar represents 100 µm. The data are presented as mean ± SD values. *n* = 8 to 10 rats in each group.* and ^#^ denote the significant differences from the control and DEN groups, respectively at *p* < 0.05 in each case.

Hepatic preneoplastic lesions in rats were induced by DEN injection, three times, which can be quantified using the established biomarker, glutathione *S*-transferase placental (GST-P)-positive foci. Accordingly, GST-P-positive foci were significantly triggered in rats injected with DEN (Figure 2). In contrast, no GST-P-positive foci were observed in the MP group, indicating that MPs were not carcinogenic to rats in this study. However, the numbers and areas of GST-P-positive foci were significantly up-regulated in DEN-induced rats following long-term exposure to MPs. These findings suggested that MPs aggravated the early stages of hepatocarcinogenesis by promoting the formation of preneoplastic lesions in DEN-induced rats.

### 3.3. MP Enhanced Cellular Proliferation in the Livers of DEN-Induced Rats

**Figure 3:**
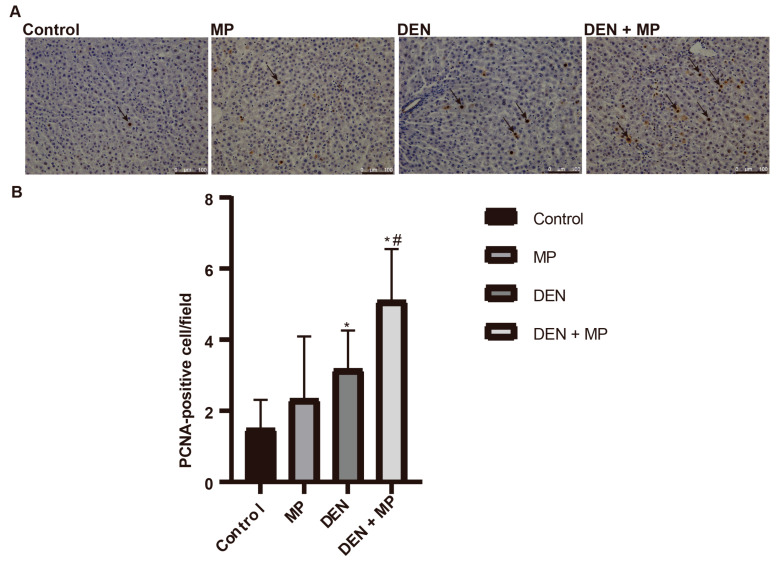
The effects of microplastics administration on cell proliferation in livers of rats. (**A**) PCNA-positive cells in rat livers, (**B**) the number of PCNA-positive cells in rat livers. MP, microplastic; DEN, diethylnitrosamine. The black arrow represents PCNA-positive hepatocytes. The scale bar represents 100 µm. The data are presented as mean ± SD values. *n* = 6 rats in each group. * and ^#^ denote significant differences from the control and DEN groups, respectively, at *p* < 0.05 in each case.

To investigate the mechanism by which MPs promote the formation of preneoplastic lesions in rat liver, cell proliferation was assessed using the marker of PCNA. The numbers of PCNA-positive cells were significantly increased in rats induced by DEN compared to those receiving normal saline (Figure 3), which is in accordance with our previous studies [36]. Notably, MP exposure alone did not affect cellular proliferation, supporting the conclusion that there were no preneoplastic lesions in livers. Furthermore, long-term exposure to MPs in DEN-induced rats enhanced the numbers of PCNA-positive cells compared to DEN-induced rats alone. These result implied that MPs exacerbated the early stages of hepatocarcinogenesis through the modulation of cell proliferation.

### 3.4. MPs Changed the Gut Microbiota Composition of DEN-Induced Rats

**Figure 4:**
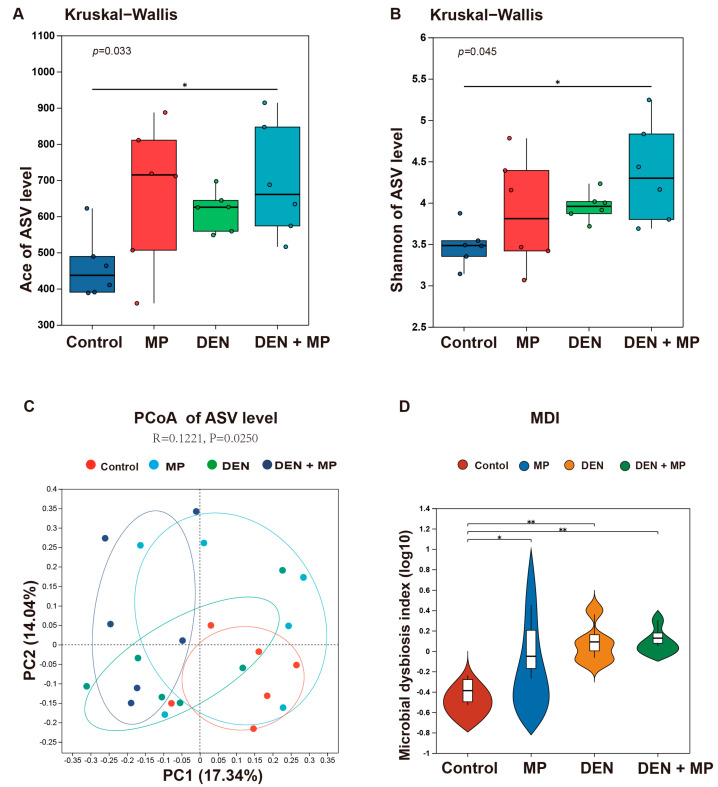
The effects of microplastics administration on the composition of rat gut microbiota. The gut microbiota alpha diversity based on (**A**) the Ace index and (**B**) the Shannon index. The beta diversity of gut microbiota based on (**C**) principal coordinate analysis and (**D**) the microbial dysbiosis index based on the amplicon sequence variants (ASVs). MP, microplastic; DEN, diethylnitrosamine; MDI, microbial dysbiosis index; PC1, principal coordinate 1; PC2, principal coordinate 2. *n* = 6 rats in each group. * and ** denote the significant differences from the control group at *p* < 0.05 and <0.01, respectively.

Accumulating evidence suggests that the gut microbiota plays an essential role in cancer development. Therefore, in this study, we assessed the profile of fecal microbiota by using 16S rDNA sequencing analysis. The samples were assessed for diversity based on the construction of the ASV set. With respect to the alpha diversity, MP administration resulted in a significantly higher Ace index in DEN-induced rats when compared to those in the Control group, indicating that MP exposure contributed to the microbiota richness of rats induced by DEN (Figure 4A). In addition, the Shannon index also showed a marked elevation in the MP + DEN group, suggesting that MP exposure enhanced microbial diversity in DEN-induced rats (Figure 4B). However, no significant changes were observed in the Ace and Shannon indexes among the other groups, despite there being an increase compared to the control. These findings indicated that the combination of MP exposure and DEN injection led to greater microbial community diversity and richness in rats.

With respect to the beta diversity, principal coordinate analysis (PCoA) at the ASV level revealed that although the gut microbiota in both the DEN and MP groups exhibited similarities to that in the Control group, some degree of deviation remained (Figure 4C). In contrast, the gut microbial composition in the MP + DEN group was distinctly separated from that of the Control group. These results suggested that the MP + DEN group had a more significant alteration of the gut microbial structure when compared to the DEN as well as the MP group. The microbial dysbiosis index (MDI) reflects the degree of microbial imbalance within the organism. Figure 4D shows a significantly higher MDI value in the treated rats when compared to the control animals, indicating that MPs as well as DEN caused microbial imbalances.

**Figure 5:**
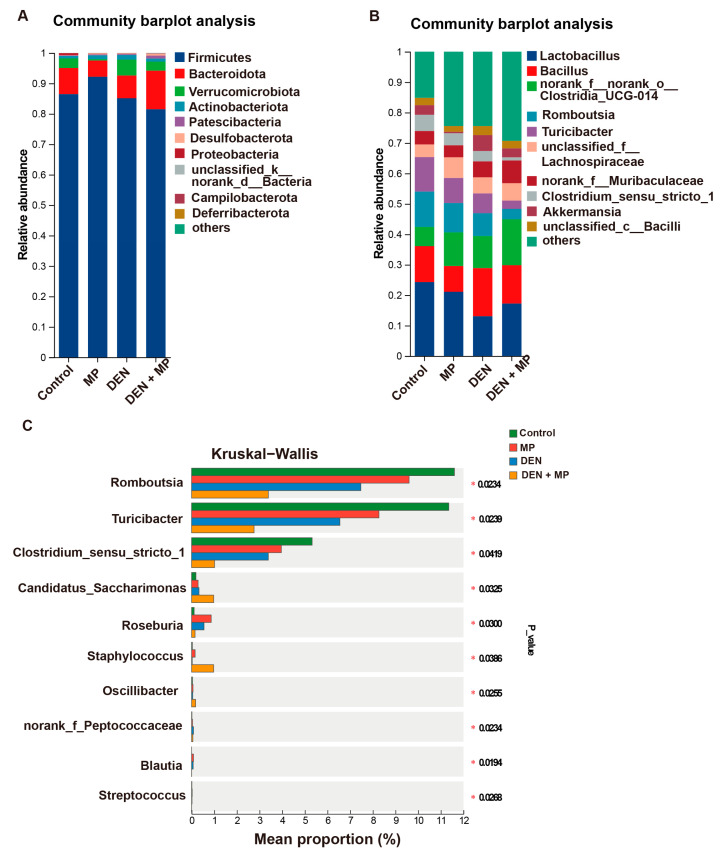
The differential microbiota composition in each group of rats in this study. The relative abundances of taxonomic compositions in each group at (**A**) the phylum level and (**B**) the genus level. (**C**) The mean proportions of differential microbiota at the genus level in each group. MP, microplastic; DEN, diethylnitrosamine. *n* = 6 rats in each group. Statistical differences are indicated as * *p* < 0.05.

The bacterial taxonomic profiles at the phylum and genus levels were further investigated (Figure 5A,B). Firmicutes, Bacteroidetes and Verrucomicrobia were the predominant phyla in the intestinal microbiota in all the animal groups. The relative abundance of Firmicutes was highly reduced in the MP + DEN group compared to the Control group, which aligned with previous studies [22]. Conversely, the relative abundance of Patescibacteria was increased in the MP + DEN group. At the genus level, the top 10 microbial genera in terms of abundance are displayed in Figure 5B, providing a clear visualization of the microbial distribution across each group. The dominant genera included *Lactobacillus*, *Bacillus norank_f__norank_o__Clostridia_UCG-014*, and *Romboutsia* in the intestinal microbiota. Furthermore, the proportions of sequences of the differential microbiota in each group are presented in Figure 5C. At the genus level, MP administration markedly increased the proportions of sequences of *Candidatus_Saccharimonas*, *Oscillibacter* and *Staphylococcus* in DEN-induced rats as compared to the Control group. Conversely, the proportions of sequences of *Clostridium_sensu_stricto_1*, *Faecalibaculum*, *Romboutsia* and *Turicibacter* were notably decreased in the MP + DEN group when compared to the Control group.

### 3.5. MPs Reshaped the Composition of SCFAs in DEN-Induced Rats

The SCFAs metabolized by the gut microbiota were determined by using GC–MS. The concentrations of various SCFAs, namely acetic, propanoic, isobutyric, butanoic, isovaleric, valeric, isohexanoic and hexanoic acids, are summarized in Table 3. Exposure to MPs resulted in a decrease in the levels of acetic, propanoic and isohexanoic acids in the feces of DEN-initiated rats compared to the Control group, while increasing the levels of isovaleric acid. In contrast, MP exposure and DEN injection alone caused fewer alterations in SCFA levels. These results implied that MPs play a crucial role in the modulation of bacterial metabolites in DEN-induced rats.

### 3.6. Correlation Analysis Between SCFAs and Gut Microbiota

**Figure 6:**
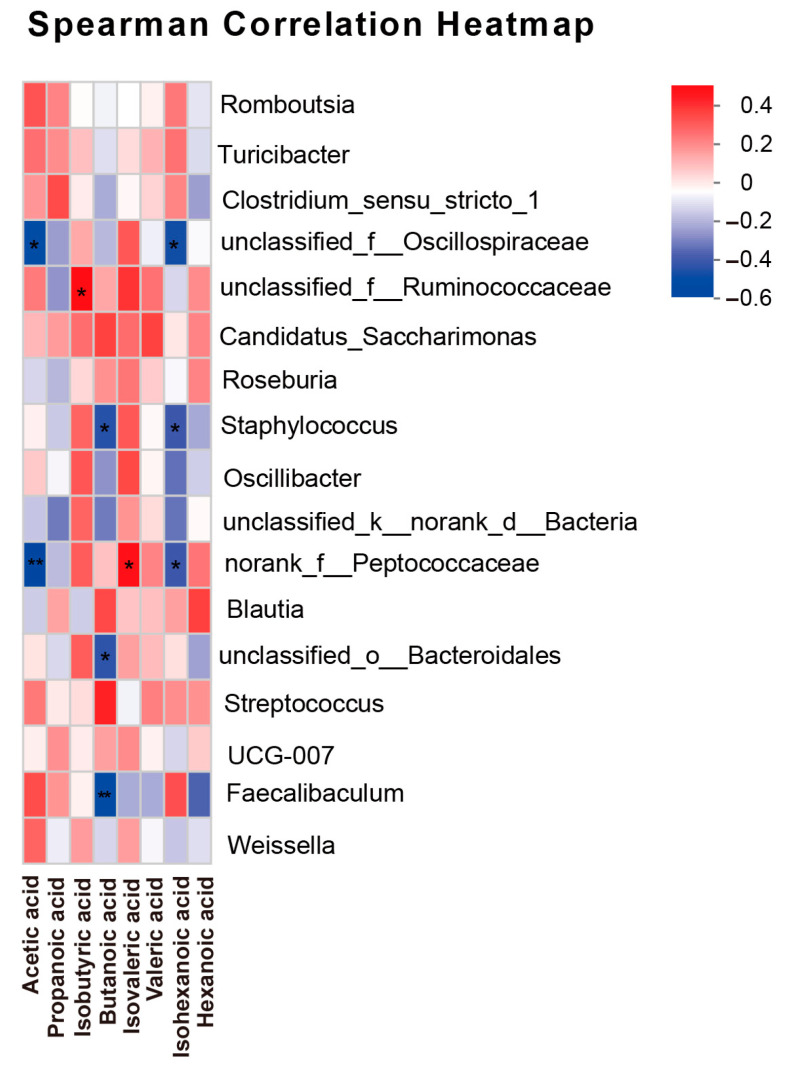
The correlations between gut microbiota and SCFAs are presented in Spearman correlation heatmap. *n* = 6 rats in each group. The colored boxes represent the levels of the correlation index. Statistical differences are indicated as * *p* < 0.05, ** *p* < 0.01.

The correlation between SCFAs and microbiota was analyzed using Spearman correlation analysis. *Staphylococcus* and *norank_f__Peptococcaceae* exhibited a notably negative correlation with the levels of acetic, butanoic and isohexanoic acids, but a positive correlation with the levels of isovaleric acid (Figure 6). Thus, there was a certain correlation between the differential microbiota and SCFAs.

## 4. Discussion

Carcinogenesis is a complex and multistep process including initiation, promotion and progression stages, all of which are influenced by environmental factors. MPs have been reported to induce liver damage in rodent models when they are exposed to these particles over periods ranging from 14 to 90 days [37]. To date, no studies have focused on the effects of long-term exposure to MPs on the early stage of hepatocarcinogenesis. Our findings indicated that long-term exposure to MPs can promote the formation of preneoplastic lesions in rats induced by DEN. This occurs primarily through the modulation of cellular proliferation as well as changes in the gut microbiota composition and the levels of SCFAs.

DEN is a major contaminant commonly found in seafoods, edible oils, fried foods, water and cigarette smoke, resulting from the nitrosation of nitrates and nitrites [38]. It has been extensively employed as a hepatocarcinogen in experimental rodents, having related histopathogenic consequences for human liver cancer. Consequently, a well-established early-stage hepatocarcinogenesis model was utilized to evaluate the health risks associated with MPs. GST-P is highly expressed during liver carcinogenesis, and it can serve as a reliable marker for experimental rat hepatocarcinogenesis [39].

General observation parameters and liver functional enzymes were utilized to evaluate the toxicity of substances. Our results indicated that long-term exposure to MPs did not cause systemic toxicity in rats. However, MP exposure in conjunction with DEN administration led to a significant decrease in these parameters, implying that MPs and DEN exhibited a synergistic adverse effect in rats. In addition, it was confirmed that the liver is more sensitive and adversely affected by MP exposure under unhealthy conditions, which aligns with the more toxic effects observed in mice co-exposed to MPs and epoxiconazole [40].

In this study, we did not find MPs be hepatocarcinogenic, as no GST-P foci were observed in rats exposed to MPs alone. MPs may act as tumor promoters by amplifying carcinogen-induced lesions, revealing a novel environmental co-factor during cancer progression. Recent studies have raised concerns regarding the potential carcinogenicity of MPs, as they have been linked to DNA damage, oxidative stress, inflammatory responses and other harmful processes associated with cancer development [41,42,43]. However, the administration of MPs did increase the number and size of GST-P-positive foci in the livers of DEN-induced rats, suggesting that these particles may modulate carcinogen metabolites. The active metabolite DEN is primarily metabolized by CYP2E1, which interacts with DNA to form certain DNA adducts, resulting in carcinogenicity [44]. Consequently, MPs have been shown to induce liver toxicity and activate the expression of xenobiotic enzymes [11,13]. Therefore, it is speculated that MPs, as xenobiotics, may enhance the toxicity of DEN by increasing the levels and/or activities of CYP2E1 in female rats.

The dysregulation of cell proliferation is one of the important factors in cancer development [45]. PCNA has proven to be a reliable biomarker for cell proliferation [46]. Rats injected with DEN exhibited upregulation of PCNA-positive cells, which was in line with our previous studies [30]. In addition, the administration of MPs significantly increased the number of PCNA-positive cells in the liver in DEN-induced rats. The results revealed that the promoting effect of MPs on the formation of preneoplastic lesions in rats was associated with the modulation of cellular proliferation. Accordingly, oral exposure to MPs for 6 weeks promoted cell proliferation, inflammatory cytokines and oxidative imbalance in the CCl_4_-induced mouse model [13], leading to liver fibrosis.

Accumulating studies have demonstrated the significant role of the gut microbiota in the carcinogenesis of HCC [17]. Exposure to MPs for 5 to 6 weeks was reported to reduce intestinal mucus secretion, alter the gut microbiota composition and induce inflammation in mice [22,47,48]. In our study, long-term exposure to MPs resulted in changes in the microbiota composition in rats. However, these changes were not significant when compared to the control group. The different observed effects of MPs on the gut microbiota may be influenced by the species or dose used in the different studies. In contrast, MP exposure combined with DEN injection led to a substantial disturbance in the microbiota structure, resulting in gut dysbiosis. The degree of microbial dysbiosis in patients with HCC has been reported to be significantly higher than that in healthy individuals [37]. Thus, alterations in the gut microbiota may increase disease susceptibility, potentially explaining why MP exposure promoted the early stages of hepatocarcinogenesis in DEN-induced rats.

At the phylum level, the proportion of Firmicutes sequences decreased in the MP + DEN group, whereas the proportion of Patescibacteria sequences increased. Lu et al. demonstrated that, in particular, the abundance of Firmicutes decreased significantly, leading to microbiota dysbiosis, and that hepatic triglyceride and total cholesterol levels declined in mice after 5 weeks of MP exposure [22]. Interestingly, Firmicutes have been linked to a reduction in the luminal abundance of SCFAs [49], which may explain the lower SCFA levels observed in the MP + DEN group. Furthermore, an increase in Firmicutes in the cecum has been reported to enhance nutrient absorption [50], potentially explaining the significant decreases observed in BWs in the MP + DEN group. In summary, Firmicutes had a foundational presence across all groups. At the genus level, several key microbiota were significantly altered in the MP + DEN group when compared to the Control group. The genera *Romboutsia* and *Turicibacter* were significantly decreased in DEN-induced rats exposed to MP. Both *Romboutsia* and *Turicibacter,* which are recognized as beneficial bacteria, have been reported to decline in the fecal microbial community of individuals with Crohn’s disease, colorectal cancer and non-alcoholic fatty liver disease [51,52,53]. On the contrary, the genera *Staphylococcus* and *Oscillibacter* showed a significant increase in DEN-induced rats exposed to MPs. These bacteria have been identified to be enriched in the early stages of HCC and colorectal cancer [54,55]. In addition, *Faecalibaculum*, which exhibits an anti-inflammatory effect (probiotics) [12], was notably decreased in the MP + DEN group [56]. Based on these findings, it is suggested that MP exposure may led to microbiota dysbiosis through the modulation of both beneficial and pathogenic bacteria in DEN-induced rats. To establish the direct causal relationship and potential immune modulation, experiments involving fecal microbiota transplantation (FMT) and the analysis of immune subsets in either hepatic or splenic tissues using flow cytometry may be necessary.

SCFAs, derived from intestinal microbiota metabolites, play a crucial role in modulating the inflammatory response, enhancing intestinal barrier function as well as taking part in the anti-carcinogenic process [57,58,59]. In our studies, when compared to the Control group, the administration of MPs or injection of DEN alone resulted in minimal changes in SCFA levels. However, a notable decrease in SCFAs, including acetic, propanoic and isohexanoic acids, was observed in the DEN + MP group when compared to the Control group. Acetic acid, while less studied compared to butyric acid, has also been shown to protect the intestinal barrier and exert anti-inflammatory effects, particularly in the context of ulcerative colitis [58]. Thus, a reduction in SCFA levels might aggravate hepatocarcinogenesis in rats exposed to MP [60]. In addition, Spearman correlation analysis showed that there was a certain correlation between the differential microbiota and SCFAs. Therefore, the combination of MP exposure and DEN injection altered the bacterial composition and its metabolites, SCFAs, potentially exacerbating the formation of preneoplastic lesions in livers.

## 5. Conclusions

We demonstrated that long-term exposure to MPs promoted hepatocarcinogenesis in rats induced by DE, via the modulation of cellular proliferation, the gut microbiota and SCFAs. However, this study primarily focused on MPs, neglecting the consideration of nanoplastics and other prevalent polymer types, as well as failing to account for complex particle morphologies. As a result, the findings may not accurately represent all the effects of exposure to MPs. This study highlights the need to evaluate the health risks of MPs in human populations.

## Figures and Tables

**Table 1:** The effects of microplastics on the characteristics of rats in this study.

Group	Initial Body Weight (g)	Final Body Weight (g)	Consumption (Per Rat Per Day)	
			Food (g)	Water (mL)
Control	101 ± 10	565 ± 43	27.4 ± 3.5	21.7 ± 5.9
MP	102 ± 7	543 ± 47	28.5 ± 1.6	22.2 ± 1.8
DEN	102 ± 14	558 ± 55	27.7 ± 2.0	21.2 ± 2.6
DEN + MP	101 ± 12	506 ± 75 *	25.4 ± 1.1	18.0 ± 0.9

The data are presented as mean ± SD values. *n* = 8 to 10 rats in each group. MP, microplastic; DEN, diethylnitrosamine. * denotes a significant difference from the control group at *p* < 0.05.

**Table 2:** The effects of microplastics on the weights of some vital organs and liver function tests in the rats.

Group	Relative Organ Weight (%)			Liver Function Test (Unit/L)	
	Liver	Spleen	Kidney	ALT	AST
Control	3.39 ± 0.30	0.18 ± 0.02	0.61 ± 0.08	54.3 ± 1.5	95.0 ± 4.2
MP	3.16 ± 0.10 *	0.18 ± 0.01	0.59 ± 0.07	63.3 ± 7.5	121.1 ± 31.9
DEN	3.24 ± 0.30	0.20 ± 0.03	0.57 ± 0.06	62.9 ± 9.2	101.7 ± 24.5
DEN + MP	3.06 ± 0.14 *	0.21 ± 0.04 *	0.55 ± 0.06	71.4 ± 9.1 *^#^	123.3 ± 21.3

The data are presented as mean ± SD values. *n* = 8 to 10 rats in each group. MP, microplastic; DEN, diethylnitrosamine; ALT, alanine aminotransferase; AST, aspartate aminotransferase. * and ^#^ denote significant differences from the control and DEN groups, respectively at *p* < 0.05 in each case.

**Table 3:** The effects of microplastics on SCFA levels in the feces of rats.

SCFA	Group			
(µg/mg Feces)	Control	MP	DEN	DEN + MP
Acetic acid	5.958 ± 0.682	5.731 ± 0.403 ^#^	4.570 ± 0.656 *	4.631 ± 0.942 *
Propanoic acid	1.438 ± 0.585	0.981 ± 0.294	0.944 ± 0.410	0.778 ± 0.273 *
Isobutyric acid	0.077 ± 0.013	0.084 ± 0.010	0.071 ± 0.020	0.101 ± 0.031 ^#^
Butanoic acid	1.485 ± 0.833	2.169 ± 1.153	1.806 ± 0.937	1.468 ± 0.837
Isovaleric acid	0.053 ± 0.010	0.065 ± 0.011	0.060 ± 0.015	0.075 ± 0.026 *
Valeric acid	0.106 ± 0.039	0.105 ± 0.016	0.091 ± 0.020	0.106 ± 0.037
Isohexanoic acid	0.005 ± 0.004	0.003 ± 0.001	0.002 ± 0.001 *	0.002 ± 0.001 *
Hexanoic acid	0.196 ± 0.098	0.294 ± 0.164	0.215 ± 0.136	0.260 ± 0.150

The data are presented as mean ± SD values with *n* = 6 rats in each group. SCFA, short-chain fatty acid; MP, microplastic; DEN, diethylnitrosamine. * and ^#^ denote significant differences from the control and DEN groups, respectively, at *p* < 0.05 in each case.

## Data Availability

The original contributions presented in the study are included in the article. Any further inquiries should be directed to the corresponding author.

## References

[1] McGivney E., Cederholm L., Barth A., Hakkarainen M., Hamacher-Barth E., Ogonowski M., Gorokhova E. (2020). Rapid physicochemical changes in microplastic induced by biofilm formation. Front. Bioeng. Biotechnol..

[2] Traversa A., Mari E., Pontecorvi P., Gerini G., Romano E., Megiorni F., Amedei A., Marchese C., Ranieri D., Ceccarelli S. (2024). Polyethylene micro/nanoplastics exposure induces epithelial-mesenchymal transition in human bronchial and alveolar epithelial cells. Int. J. Mol. Sci..

[3] Zitouni N., Bousserrhine N., Belbekhouche S., Missawi O., Alphonse V., Boughatass I., Banni M. (2020). First report on the presence of small microplastics (≤3 μm) in tissue of the commercial fish Serranus scriba (Linnaeus. 1758) from Tunisian coasts and associated cellular alterations. Environ. Pollut..

[4] Thompson R.C., Courtene-Jones W., Boucher J., Pahl S., Raubenheimer K., Koelmans A.A. (2024). Twenty years of microplastic pollution research-what have we learned?. Science.

[5] van der Laan L.J.W., Bosker T., Peijnenburg W. (2023). Deciphering potential implications of dietary microplastics for human health. Nat. Rev. Gastroenterol. Hepatol..

[6] Yang J.D., Hainaut P., Gores G.J., Amadou A., Plymoth A., Roberts L.R. (2019). A global view of hepatocellular carcinoma: Trends, risk, prevention and management. Nat. Rev. Gastroenterol. Hepatol..

[7] Bray F., Laversanne M., Sung H., Ferlay J., Siegel R.L., Soerjomataram I., Jemal A. (2024). Global cancer statistics 2022: GLOBOCAN estimates of incidence and mortality worldwide for 36 cancers in 185 countries. CA A Cancer J. Clin..

[8] Huang H., Hou J., Yu C., Wei F., Xi B. (2024). Microplastics exacerbate tissue damage and promote carcinogenesis following liver infection in mice. Ecotoxicol. Environ. Saf..

[9] Horvatits T., Tamminga M., Liu B., Sebode M., Carambia A., Fischer L., Püschel K., Huber S., Fischer E.K.J.E. (2022). Microplastics detected in cirrhotic liver tissue. EBioMedicine.

[10] Pontecorvi P., Ceccarelli S., Cece F., Camero S., Lotti L.V., Niccolai E., Nannini G., Gerini G., Anastasiadou E., Scialis E.S. (2023). Assessing the impact of polyethylene nano/microplastic exposure on human vaginal keratinocytes. Int. J. Mol. Sci..

[11] Cheng W., Li X., Zhou Y., Yu H., Xie Y., Guo H., Wang H., Li Y., Feng Y., Wang Y. (2022). Polystyrene microplastics induce hepatotoxicity and disrupt lipid metabolism in the liver organoids. Sci. Total Environ..

[12] Zhang K., Yang J., Chen L., He J., Qu D., Zhang Z., Liu Y., Li X., Liu J., Li J. (2023). Gut microbiota participates in polystyrene microplastics-induced hepatic injuries by modulating the gut-liver axis. ACS Nano.

[13] Djouina M., Waxin C., Dubuquoy L., Launay D., Vignal C., Body-Malapel M. (2023). Oral exposure to polyethylene microplastics induces inflammatory and metabolic changes and promotes fibrosis in mouse liver. Ecotoxicol. Environ. Saf..

[14] Liu J., Zhang L., Xu F., Meng S., Li H., Song Y. (2022). Polystyrene microplastics postpone APAP-induced liver injury through impeding macrophage polarization. Toxics.

[15] Wen S., Zhao Y., Liu S., Chen Y., Yuan H., Xu H. (2022). Polystyrene microplastics exacerbated liver injury from cyclophosphamide in mice: Insight into gut microbiota. Sci. Total Environ..

[16] Acharya C., Sahingur S.E., Bajaj J.S. (2017). Microbiota, cirrhosis, and the emerging oral-gut-liver axis. JCI Insight.

[17] Zhou A., Tang L., Zeng S., Lei Y., Yang S., Tang B. (2020). Gut microbiota: A new piece in understanding hepatocarcinogenesis. Cancer Lett..

[18] Petersen C., Round J.L. (2014). Defining dysbiosis and its influence on host immunity and disease. Cell. Microbiol..

[19] Ni J., Huang R., Zhou H., Xu X., Li Y., Cao P., Zhong K., Ge M., Chen X., Hou B. (2019). Analysis of the relationship between the degree of dysbiosis in gut microbiota and prognosis at different stages of primary hepatocellular carcinoma. Front. Microbiol..

[20] Wan M.L.Y., El-Nezami H. (2018). Targeting gut microbiota in hepatocellular carcinoma: Probiotics as a novel therapy. Hepatobiliary Surg. Nutr..

[21] Kalyanaraman B., Cheng G., Hardy M. (2024). The role of short-chain fatty acids in cancer prevention and cancer treatment. Arch. Biochem. Biophys..

[22] Lu L., Wan Z., Luo T., Fu Z., Jin Y. (2018). Polystyrene microplastics induce gut microbiota dysbiosis and hepatic lipid metabolism disorder in mice. Sci. Total Environ..

[23] Dokkaew A., Punvittayagul C., Insuan O., Limtrakul Dejkriengkraikul P., Wongpoomchai R. (2019). Protective effects of defatted sticky rice bran extracts on the early stages of hepatocarcinogenesis in rats. Molecules.

[24] Zhao B., Rehati P., Yang Z., Cai Z., Guo C., Li Y. (2024). The potential toxicity of microplastics on human health. Sci. Total Environ..

[25] Gouin T., Ellis-Hutchings R., Thornton Hampton L.M., Lemieux C.L., Wright S.L. (2022). Screening and prioritization of nano- and microplastic particle toxicity studies for evaluating human health risks—Development and application of a toxicity study assessment tool. Microplastics Nanoplastics.

[26] Khuanphram N., Taya S., Kongtawelert P., Wongpoomchai R. (2021). Sesame extract promotes chemopreventive effect of hesperidin on early phase of diethylnitrosamine-initiated hepatocarcinogenesis in rats. Pharmaceutics.

[27] Shi C., Han X., Guo W., Wu Q., Yang X., Wang Y., Tang G., Wang S., Wang Z., Liu Y. (2022). Disturbed gut-liver axis indicating oral exposure to polystyrene microplastic potentially increases the risk of insulin resistance. Environ. Int..

[28] Rafiee M., Dargahi L., Eslami A., Beirami E., Jahangiri-Rad M., Sabour S., Amereh F. (2018). Neurobehavioral assessment of rats exposed to pristine polystyrene nanoplastics upon oral exposure. Chemosphere.

[29] Deng Y., Chen H., Huang Y., Zhang Y., Ren H., Fang M., Wang Q., Chen W., Hale R.C., Galloway T.S. (2022). Long-term exposure to environmentally relevant doses of large polystyrene microplastics disturbs lipid homeostasis via bowel function interference. Environ. Sci. Technol..

[30] Guo H., Punvittayagul C., Vachiraarunwong A., Phannasorn W., Wongpoomchai R. (2022). Cancer chemopreventive potential of cooked glutinous purple rice on the early stages of hepatocarcinogenesis in rats. Front. Nutr..

[31] Zeng H., He S., Xiong Z., Su J., Wang Y., Zheng B., Zhang Y. (2023). Gut microbiota-metabolic axis insight into the hyperlipidemic effect of lotus seed resistant starch in hyperlipidemic mice. Carbohydr. Polym..

[32] Phannasorn W., Pharapirom A., Thiennimitr P., Guo H., Ketnawa S., Wongpoomchai R. (2022). Enriched riceberry bran oil exerts chemopreventive properties through anti-inflammation and alteration of gut microbiota in carcinogen-induced liver and colon carcinogenesis in rats. Cancers.

[33] Zuri G., Karanasiou A., Lacorte S. (2023). Microplastics: Human exposure assessment through air, water, and food. Environ. Int..

[34] Zhou Q., Deng J., Pan X., Meng D., Zhu Y., Bai Y., Shi C., Duan Y., Wang T., Li X. (2022). Gut microbiome mediates the protective effects of exercise after myocardial infarction. Microbiome.

[35] Zhang K., Chen L., Yang J., Liu J., Li J., Liu Y., Li X., Chen L., Hsu C., Zeng J. (2023). Gut microbiota-derived short-chain fatty acids ameliorate methamphetamine-induced depression- and anxiety-like behaviors in a Sigmar-1 receptor-dependent manner. Acta. Pharm. Sin. B..

[36] Taya S., Dissook S., Ruangsuriya J., Yodkeeree S., Boonyapranai K., Chewonarin T., Wongpoomchai R. (2024). Thai fermented soybean (Thua-Nao) prevents early stages of colorectal carcinogenesis induced by diethylnitrosamine and 1,2-dimethylhydrazine through modulations of cell proliferation and gut microbiota in rats. Nutrients.

[37] Tang K.H.D., Li R., Li Z., Wang D. (2024). Health risk of human exposure to microplastics: A review. Environ. Chem. Lett..

[38] Park J.E., Seo J.E., Lee J.Y., Kwon H. (2015). Distribution of seven N-nitrosamines in food. Toxicol. Res..

[39] Sato K. (1989). Glutathione transferases as markers of preneoplasia and neoplasia. Adv. Cancer Res..

[40] Sun W., Yan S., Meng Z., Tian S., Jia M., Huang S., Wang Y., Zhou Z., Diao J., Zhu W. (2022). Combined ingestion of polystyrene microplastics and epoxiconazole increases health risk to mice: Based on their synergistic bioaccumulation in vivo. Environ. Int..

[41] Barguilla I., Domenech J., Ballesteros S., Rubio L., Marcos R., Hernández A. (2022). Long-term exposure to nanoplastics alters molecular and functional traits related to the carcinogenic process. J. Hazard. Mater..

[42] Kumar N., Lamba M., Pachar A.K., Yadav S., Acharya A. (2024). Microplastics—A growing concern as carcinogens in cancer etiology: Emphasis on biochemical and molecular mechanisms. Cell Biochem. Biophys..

[43] Dzierżyński E., Gawlik P.J., Puźniak D., Flieger W., Jóźwik K., Teresiński G., Forma A., Wdowiak P., Baj J., Flieger J. (2024). Microplastics in the human body: Exposure, detection, and risk of carcinogenesis: A state-of-the-art review. Cancers.

[44] Verna L., Whysner J., Williams G.M. (1996). N-nitrosodiethylamine mechanistic data and risk assessment: Bioactivation, DNA-adduct formation, mutagenicity, and tumor initiation. Pharmacol. Ther..

[45] Evan G.I., Vousden K.H. (2001). Proliferation, cell cycle and apoptosis in cancer. Nature.

[46] Bologna-Molina R., Mosqueda-Taylor A., Molina-Frechero N., Mori-Estevez A.D., Sánchez-Acuña G. (2013). Comparison of the value of PCNA and Ki-67 as markers of cell proliferation in ameloblastic tumors. Med. Oral Patol. Oral Cir. Bucal.

[47] Jin Y., Lu L., Tu W., Luo T., Fu Z. (2019). Impacts of polystyrene microplastic on the gut barrier, microbiota and metabolism of mice. Sci. Total Environ..

[48] Li B., Ding Y., Cheng X., Sheng D., Xu Z., Rong Q., Wu Y., Zhao H., Ji X., Zhang Y. (2020). Polyethylene microplastics affect the distribution of gut microbiota and inflammation development in mice. Chemosphere.

[49] Libertucci J., Dutta U., Kaur S., Jury J., Rossi L., Fontes M.E., Shajib M.S., Khan W.I., Surette M.G., Verdu E.F. (2018). Inflammation-related differences in mucosa-associated microbiota and intestinal barrier function in colonic Crohn’s disease. Am. J. Physiol..

[50] Ley R.E., Bäckhed F., Turnbaugh P., Lozupone C.A., Knight R.D., Gordon J.I. (2005). Obesity alters gut microbial ecology. Proc. Natl. Acad. Sci. USA.

[51] Takahashi K., Nishida A., Fujimoto T., Fujii M., Shioya M., Imaeda H., Inatomi O., Bamba S., Sugimoto M., Andoh A. (2016). Reduced abundance of butyrate-producing bacteria species in the fecal microbial community in Crohn’s disease. Digestion.

[52] Yun Y., Kim H.N., Lee E.J., Ryu S., Chang Y., Shin H., Kim H.L., Kim T.H., Yoo K., Kim H.Y. (2019). Fecal and blood microbiota profiles and presence of nonalcoholic fatty liver disease in obese versus lean subjects. PLoS ONE.

[53] Sanapareddy N., Legge R.M., Jovov B., McCoy A., Burcal L., Araujo-Perez F., Randall T.A., Galanko J., Benson A., Sandler R.S. (2012). Increased rectal microbial richness is associated with the presence of colorectal adenomas in humans. ISME J..

[54] Ren Z., Li A., Jiang J., Zhou L., Yu Z., Lu H., Xie H., Chen X., Shao L., Zhang R. (2019). Gut microbiome analysis as a tool towards targeted non-invasive biomarkers for early hepatocellular carcinoma. Gut.

[55] Flemer B., Lynch D.B., Brown J.M., Jeffery I.B., Ryan F.J., Claesson M.J., O’Riordain M., Shanahan F., O’Toole P.W. (2017). Tumour-associated and non-tumour-associated microbiota in colorectal cancer. Gut.

[56] Lopez-Siles M., Duncan S.H., Garcia-Gil L.J., Martinez-Medina M. (2017). Faecalibacterium prausnitzii: From microbiology to diagnostics and prognostics. ISME J..

[57] Liu P., Wang Y., Yang G., Zhang Q., Meng L., Xin Y., Jiang X. (2021). The role of short-chain fatty acids in intestinal barrier function, inflammation, oxidative stress, and colonic carcinogenesis. Pharmacol. Res..

[58] Deleu S., Arnauts K., Deprez L., Machiels K., Ferrante M., Huys G.R.B., Thevelein J.M., Raes J., Vermeire S. (2023). High acetate concentration protects intestinal barrier and exerts anti-inflammatory effects in organoid-derived epithelial monolayer cultures from patients with Ulcerative Colitis. Int. J. Mol. Sci..

[59] Hodgkinson K., El Abbar F., Dobranowski P., Manoogian J., Butcher J., Figeys D., Mack D., Stintzi A. (2023). Butyrate’s role in human health and the current progress towards its clinical application to treat gastrointestinal disease. Clin. Nutr..

[60] Feitelson M.A., Arzumanyan A., Medhat A., Spector I. (2023). Short-chain fatty acids in cancer pathogenesis. Cancer Metastasis Rev..

